# Comparison of Titanium and Bioresorbable Plates in “A” Shape Plate Properties—Finite Element Analysis

**DOI:** 10.3390/ma12071110

**Published:** 2019-04-03

**Authors:** Rafał Zieliński, Marcin Kozakiewicz, Jacek Świniarski

**Affiliations:** 1 Department of Maxillofacial Surgery, Medical University of Lodz, 1st Haller Plac, 90-647 Lodz, Poland; qed@op.pl; 2 Department of Strength of Materials and Structures, Technical University of Lodz, Stefanowskiego 1/15, 90-924 Lodz, Poland; jacek.swiniarski@p.lodz.pl

**Keywords:** mandible condylar fractures, surgical treatment, titanium, PLLA, finite element analysis

## Abstract

(1) Background: The main disadvantage of rigid fracture fixation is remain material after healing period. Implementation of resorbable plates prevents issues resulting from left plates. The aim of this study is to compare the usage of bioresorbable and titanium “A” shape condyle plate in condylar fractures. (2) Methods: Thickness of 1.0 mm, height of 31 mm, and width of 19 mm polylactic acid (PLLA) and titanium “A” shape plate with 2.0 mm-wide connecting bar and 9 holes were tested with finite element analysis in high right condylar neck fracture. (3) Results: On bone surface the highest stress is on the anterior bridge around first hole (approx. 100 MPa). The highest stress on screws is located in the first screw around plate in the anterior bridge and is greater in titanium (150 MPa) than PLLA (114 MPa). (4) Conclusion: Pressure on bone in PLLA osteosynthesis is two times higher than in titanium fixation. On small areas where pressure on bone is too high it causes local bone degradation around the fracture and may delay the healing process or make it impossible. Fixation by PLLA is such flexible that bone edges slide and twist what may lead to degradation of callus.

## 1. Introduction

Open reduction internal fixation is the method of choice in mandibular condyle fractures [1,2,3]. Although it demands the surgical skills because of the risk of facial palsy. Titanium alloys are still gold standard in terms of osteosynthesis fixation systems, however, bioresorbable plates and screws are also used in condyle fractures. The main disadvantage of metal plates is when the necessity of removal of osteosynthesis material appears (especially extraoral scars or facial nerve palsy) [4]. Loosening of metal plates or screws which might not be visible on radiographs also demand removal [5,6,7]. According to the literature there are a few reasons where removing of metal plates should be performed: metallosis, corrosion, thermal dysaesthesia, difficulties with future radiological diagnosis, malpositioning, and the migration of osteosynthesis material, particularly in craniofacial surgery [8,9].

Insertion of resorbable screws demand tapping because of the mechanical properties of the material. In long screws there is high risk of fracture of the neck of the screw. Cutting of the threads take much time and any other additional manipulation is linked with the possibility of displacement of condylar fragments. In the “bone welding” method, screws are not inserted such as self-tapping screws but resorbable pins are placed by ultrasound activation [10,11,12,13]. The pin is put into the drilled hole and link with osseointegrate with spongy bone.

There are various ways how to perform rigid internal fracture fixation in the condylar area (base, middle, high neck, and head) and it significantly changed in the last decade. To our knowledge, there are no published reports concerning what fixation system and which material provide functionally stable fixation in fractures of the condylar neck of the mandible.

The aim of the study is to compare by means of finite element analysis (FEA) one of the bioresorbable type of “A” shape condyle plate and screws made of polylactic acid (PLLA) versus titanium grade 5 alloy (Ti6Al4V).

## 2. Materials and Methods

### 2.1. The Plate

**Figure 1:**
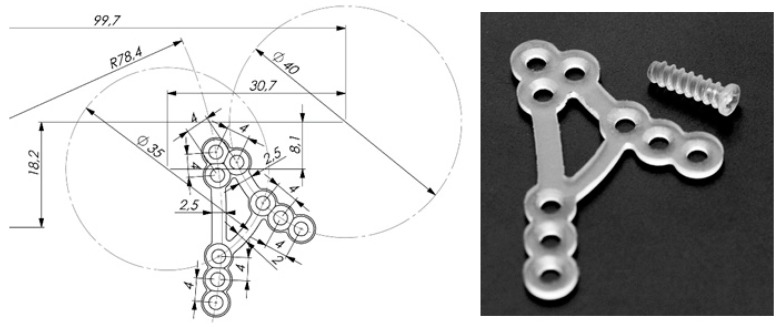
Right side shows the photo of resorbable polylactic acid (PLLA) A-shape right plate and screw whereas on the left side sketch with the dimensions. The height and the width of “A” shape plate are 31 mm and 19 mm respectively whereas the thickness is 1.0 mm. The width of the bridge is 2.5 mm. There are three linearly located holes on inferior tails of the bridges. Superiorly, the bridges converge and has triangular 3-hole group. Two tails are connected by means of 2.0 mm wide bar.

**Figure 2:**
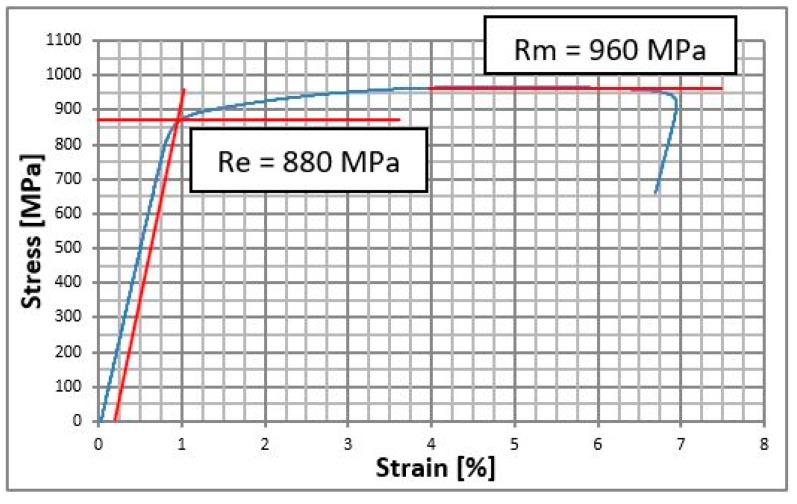
Stress strain curve for the Ti-6Al-4V Grade 5 titanium alloy.

Recently developed resorbable plate (ChM company, Lewickie, Poland, www.chm.eu) has been tested. Manufacturer applied their own new PLLA polymer. Figure 1 shows the design of A-shape condylar plate (ACP) made of resorbable material. The height and the width of the PLLA plate are 31 mm and 19 mm, respectively, whereas the thickness is 1.0 mm. The first bridge of ACP is parallel to posterior border of the mandibular ramus (compression area) and the second is parallel to the sigmoid notch (traction area). The width of the bridge is 2.5 mm. Bridges of ACP are strictly designed according to known compression and traction lines in the mandibular ramus (Figure 2) [14]. There are three linearly located holes on inferior tails of the bridges. Superiorly, the bridges converge and has triangular 3 hole group. Two tails are connected by means of 2.0 mm wide bar. The connecting bar has been designed according to compression line of condylar neck [15].

### 2.2. Material Properties

Mechanical properties have been implemented to calculations are following: Young modulus for Ti-6Al-4V Grade 5 is 140 GPa, Poisson coefficient was 0.3, Yield stress Re = 880 MPa, ultimate tensile stress Rm = 960 MPa, plasticity modulus Ep = 1.4 GPa. For polymer material PLLA: Young modulus 3.5 GPa, Poisson coefficient was 0.35 [16], durability of stretch from 195 MPA to 240 MPa. For mandible material following data were adopted: Young modulus 14 GPa, Poisson coefficient 0.28. Bone is the material that is not described by Hooke’s law and it does not have neither linearly elastic characteristic nor plasticity modulus.

Static tensile (Instron 5989 with extensometer, Zwick/Roell, Ulm, Germany) test according to ISO 6892-1 [16] was performed in order to verify the properties of Ti-6Al-4V grade 5. The test results are shown in the graph above (Figure 2). Properties of bone and PLLA have been set on the basis of the literature [17].

### 2.3. Finite Element Analysis

Finite element models are helpful for simulating endurance of 3D complex models [18,19,20].

In the study FEM (Finite Element Method) was used for the comparison between bone end movements just after the open rigid internal fixation by titanium and PLLA plates.

During stretching bone is nonlinearly elastic and after achieving endurance on stretching or squeezing it breaks. In real life bone is not isotropic material but orthotropic, with precise mechanical properties that are different in each layer and maximal load forces in bone depends on direction of loading. Mathematical methods especially for orthotropy are linearly-elastic till the moment of bone destruction. There are several restrictions that in significant way influence on FEM results. Drawing conclusions are made on the basis of the FEM researcher and modelling of biomechanical structure and degree of its simplicity was the basis for the researchers’ team. Discussion about FEM results presented as comparison between titanium osteosynthesis and PLLA for the same one.

Authors of this study know advantages and disadvantages of finite element method and restrictions of this method. The software for preparing FEM and simulation was Ansys version 18.2 (Ansys Inc., Canonsburg, PA, USA, www.ansys.com). The proper name of used element is SOLID187 individual name for ANSYS. Users of other software might not know this name whereas tetrahedron tet10 is used for other systems. Mathematical model FEM was solved with material and construction unlinearity using significant displacements. Total size of the quest was DOF (degree of freedom) = 4350480.

### 2.4. Models

**Figure 3:**
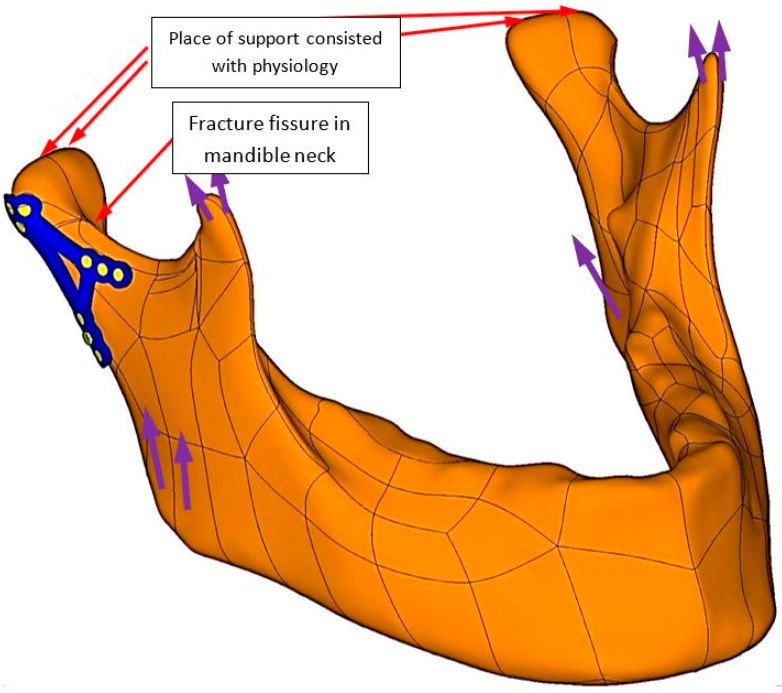
3D model with plate fixation of high neck condylar fracture (red indicators). Fixation has its physiological support points and it was loaded by muscles forces (purple indicators). In the Figure 3 purple indicators have been described in the literature [20].

In the Figure 3 mandible 3D model was shown with fixation bone ends in high fracture of condylar neck. Osteosynthesis was designed according to physiology and loaded by forces (purple colour shows forces from muscles) direction and value described by Ramos [21].

In real fractures ragged contours are observed, whereas mathematical model of friction refers to flat surfaces that irregularities are not designed and surface is described as coefficient of friction. In the study to simulate surface degradation by fracture not physical, a (extremely high) coefficient of friction of μ = 0.8 was adopted. The highest coefficient of friction that is gained in machine construction is μ = 0.65, found in brake blocks in aircraft.

The plates were fixed with mandible on basis of continuous displacement. There is initial state of strain between screws and bone because of screw fixation. Contact situation can decrease the stiffness of the connection screw–bone in order to save some time on calculations authors have decided to eliminate the threads on the screw and perform continuous displacements between simplified model of the screw and bone.

## 3. Results

Authors compared the state of bone ends in contact, pressure in contact, displacement in contact, dimension of fissure in contact and comparison maximal equivalent stress distribution in mandible and in osseofixation system.

**Figure 4:**
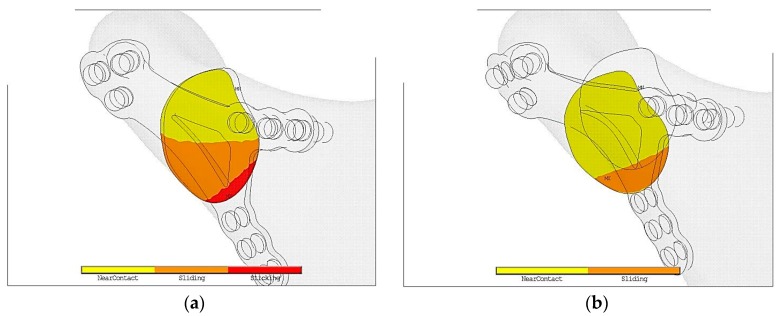
Contact status just after following plate fixation in the maximal loading according to the literature [16]: (**a**) Ti-6Al-4V plate fixation—insignificant displacement at the expense of high contact surface of bone ends during loading thanks to which appearance of minimal twisting of bone ends appeared, (**b**) PLLA plate fixation—significant displacement of bone ends thanks to which high twist appeared.

In the Figure 4a in titanium fixation half of contact area is marked in yellow color. It means that in such a fixation contact is open. Open contact is the reason of so called mechanical silence which might block healing of fracture. However, it is possible that in such conditions collagen mesh may appear and vessels from the region may grow. As a result callus becomes calcified.

Orange color shows bone in contact but it is slip so there is possibility of displacement. Cutting forces cause displacement of bone ends and also destroy the gentle collagen mesh preventing calcification, that would never calcified. As a result bone does not heal properly.

**Figure 5:**
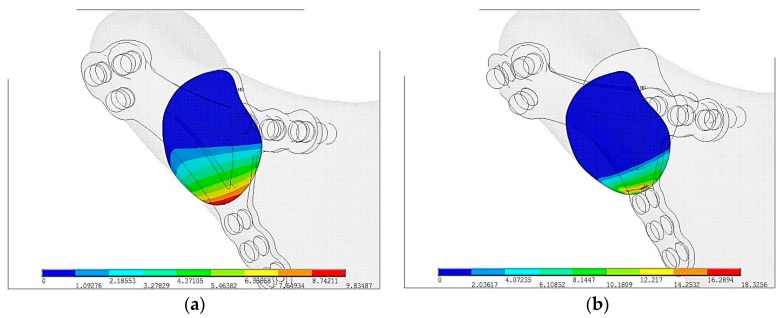
Displacements of pressure in mandibular bone ends just after plate fixation in the maximal loading according to the literature [16]: (**a**) Ti-6Al-4V plate fixation, p_max_ = 9.8 MPa, (**b**) PLLA plate fixation, p_max_ = 18.3 MPa.

Red color describes condition where both parts of bones do not displace to each other. As Figure 5b proves in PLLA fixation is much worse environment that obstruct the healing process in comparison to titanium alloy plate.

As Figure 5a shows surface pressure in contact p_max_ = 9.8 MPa are almost twice lower than in PLLA p_max_ = 18.3 MPa but the area of contact is significantly higher in plate made in Ti-6Al-4V than in PLLA, shown on Figure 5b. Greater area of surface pressure makes healing process faster and as a result it proves osseofixation stiffness.

**Figure 6:**
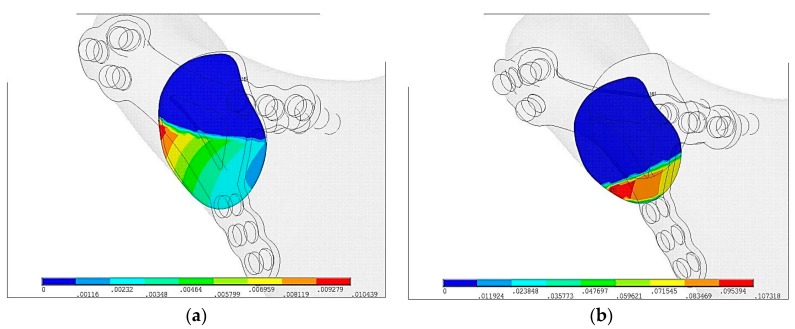
Displacements of bone ends just after plate fixation in the maximal loading according to the literature [16]: (**a**) Ti-6Al-4V plate fixation, u_max_ = 0.01 mm, (**b**) PLLA plate fixation, u_max_ = 0.1 mm.

In Figure 6 displacements of bone ends in fracture plane are shown. Blue area shows the fissure and relative displacements in plane is indeterminable. Displacement on contact surface in Ti-6Al-4V plate is u_max_ = 0.01 mm and is 10 times lower than PLLA plate u_max_ = 0.1 mm.

**Figure 7:**
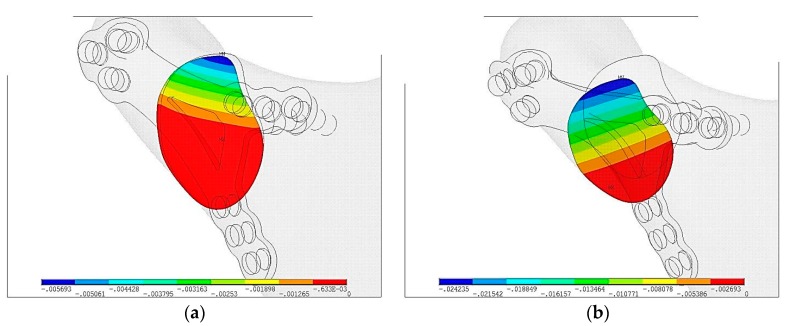
The size of the fissure between bone ends just after plate fixation in the maximal loading according to the literature [16]: (**a**) Ti-6Al-4V plate fixation, g_max_ = 0.0057 mm, (**b**) PLLA plate fixation, g_max_ = 0.024 mm.

In Figure 7 size of fissures in plate fixation were compared. Red color means that surface is in contact. Colors from orange to blue means fissure is getting wider. In Ti-6Al-4V plate the widest fissure is g_max_ = 0.0057 mm whereas in PLLA plate fixation it is g_max_ = 0.024 mm.

**Figure 8:**
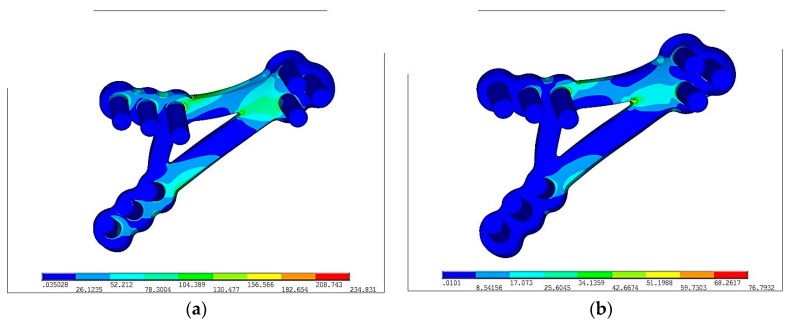
Equivalent stress distribution on plates according to von Mises hypothesis following plate fixation in the maximal loading according to the literature [16]: (**a**) Ti-6Al-4V plate fixation, **σ**_red max_ = 234 MPa, (**b**) PLLA plate fixation, **σ**_red max_ = 76 MPa; Colors from blue to red means stresses from 0 to maximum.

Color from blue to red means increasing stresses from 0 to maximum marked on red. In the Figure 8, reduced stresses distribution according to von Mises on PLLA plate with fixing screws made in Ti-6Al-4V and PLLA were the following: **σ**_red max_ = 234 MPa and **σ**_red max_ = 76 MPa respectively. Reduced stresses are 4 times lower in PLLA screws than in Ti-6Al-4V grade 5 plate. The fact of paramount importance is that Young modulus for Ti-6Al-4V grade 5 is forty times higher than PLLA. Plate made in more flexible material (PLLA) is prone to higher deformation than Ti-6Al-4V. Titanium alloy grade 5 allow for higher loading forces and make the osseofixation much rigid. Thus titanium alloy material make the healing process easier because of the immobilization of the bone ends. In any of discussed situation, yield stress was not exceeded.

**Figure 9:**
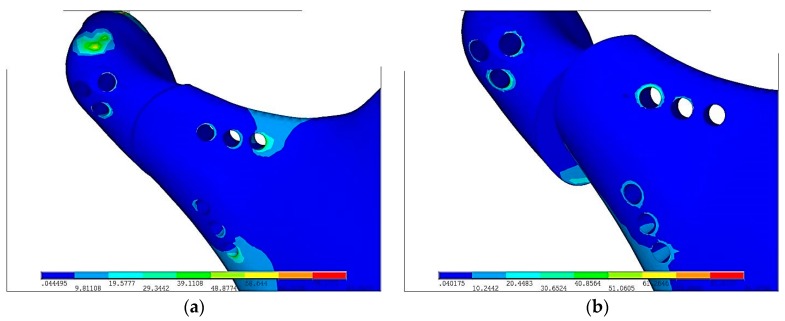
Equivalent stress distribution in mandibular condyle according to von Mises hypothesis just after plate fixation in the maximal loading according to the literature [16]: (**a**) Ti-6Al-4V plate fixation, **σ**_red max_ = 87MPa, (**b**) PLLa plate fixation, **σ**_red max_ = 91MPa; Colors from blue to red means increasing stresses from 0 to maximum.

In the Figure 9, reduced stresses distribution according to von Mises on Ti-6Al-4V and PLLA plate were **σ**_red max_ = 87 MPa and PLLA—**σ**_red max_ = 91 MPa, respectively. In both cases stresses are comparable. In order to precise the difference of stresses on bone after fixation, strain has been reduced to 30 MPa.

**Figure 10:**
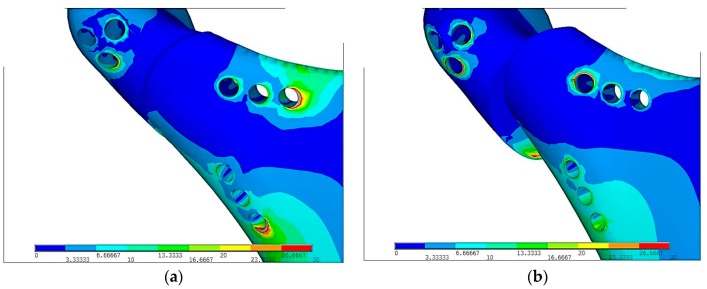
Reduced stress distribution in mandibular condyle according to von Mises hypothesis just after plate fixation in the maximal loading according to the literature [16]: (**a**) Ti-6Al-4V plate fixation, (**b**) PLLA plate fixation; Stresses have been reduced to **σ**_red max_ = 30 MPa.

Grey color on Figure 10 shows strain above the scale. Comparing 10(a) and 10(b) figures Ti-6Al-4V plate and screws indicates the center of strains is around last screws in lower part of mandible. In PLLA plate the highest stress is around the hole that is located near the fracture. This location is at risk during surgical fixation because of secondary fractures during plate fixation that restricts possibility of PLLA usage.

## 4. Discussion

Bioresorbability is the most desire feature and titanium alloy does not have it. After fixation bone ends osseointegrate in the proper way when the material is useless. Bioresorbable fixation decomposes and the patient’s state will be the same as before the surgical procedure “restitution ad integrum”. Titanium alloys that are commonly used in osteosynthesis are deprived of such a feature. Implantation of titanium fixation stays with a patient till the end of the life unless it was screwed out by the surgeon. In some cases especially when a patient grows implantation of plate and screws demand removal immediately after bone healing. It required second surgery made in postsurgical scar and facial nerve branch re-preparation (Figure 1). That may lead to permanent facial nerve palsy, thus bioresorbable materials have found its application in maxillofacial surgery.

Surgeons often during the operation in the most cases tighten in the screws without dynamometer key. It might be assumed that every single screw might be screwed with different torque and as a result stress on every screw is different. This matter has been omitted in order to simplify the procedure as well. Authors have focused on modelling of the contact of the bone ends just after the fracture and comparison of fixation materials taking into consideration only contact between bone ends. On the basis of computed tomography 3D model of edentulous mandible has been designed. Loading on the model is described as loading area transformed to the notches. Supports in temporomandibular joint have been defined as superior notch and linked on the real contact surface by Rigid Body Element (RBE). Such modelling ensures free rotation around joint. On the surface where earlier geometrically teeth have been extracted, degree of freedom are linked with the process of biting (in the direction perpendicular to the surface where teeth have been extracted).

Just after fixation, a patient who fulfils diet precautions and low forces on chewing is able to function almost normally. However, if a surgeon wants to achieve total biodegradability of fixation, then PLLA is the material of choice. The most important factor regarding durability is to perform stresses less than yield stress in order to prevent from irreversible deformation of screws and plates. That is why maxillofacial surgeon asks patients not to overload jaws regarding biting tough food especially after the surgery. Process of making new bone takes about 2 weeks and after this time new bone starts to load forces [22]. By using PLLA materials time of compulsory low forces chewing lengthens from a few days to a few months.

Plates made in PLLA or PLGA are biodegradable in comparison to Ti-6Al-4V. In terms of pressure in fracture, displacement, strain condition Ti-6Al-4V is superior to polymer/composite materials apart from biodegradability. PLLA osteosynthesis indicates that in comparison to titanium alloy fixation contact surface plate with slightly sliding between bones and plates is only 30%. Moreover, displacement between bones has occurred. In the Figure 3 it is easy to notice that in titanium fixation, so called mechanical silence occurs on half of the area after fracture whereas in case of PLLA plate only 20% of the area is in mechanical silence thus bone healing is not possible. Comparing PLLA to titanium alloy small area of concentration of the forces were observed. That is why pressure on bone in PLLA osteosynthesis is twice higher than in titanium fixation. On small areas where pressure on bone is too high it may cause local bone degradation around the fracture (i.e., bone atrophy by compression) and it might be the reason of delay in the healing process or make it impossible. The same conclusion might be assumed by observing cutting forces in the fracture shown in the Figure 4. The higher forces the higher displacement in fracture plane. In significant displacement of bone fragments in fracture fissure, collagen fibres tear and inhibit the healing process (Figure 5). Using PLLA plate has another negative feature if it comes to displacement in fracture plane. Osteosynthesis by means of PLLA is such flexible that strong biting might cause opening of fracture fissure resulting in sliding and twisting that lead to degradation of callus. Using PLLA plates require additional immobilization such as intermaxillary traction or liquid diet. Fixation cannot be overloaded otherwise bone healing process was inhibited.
